# Supplementary material for: Composite environmental indices—a case of rickety rankings
Source: PeerJ. 2023 Dec 11;11:e16325. doi: 10.7717/peerj.16325 (PMC10720475; doi:10.7717/peerj.16325)
Supplement: Supplemental Information 2 — Note. **Correlation is significant at the 0.01 level (2 tailed) [file peerj-11-16325-s002.docx]

**Summary Results of Kendall’s τ Examining Rank Correlations for 10 Iterations of the Environmental Sustainability Index (ESI) and Environmental Performance Index (EPI) (*n* = 94 countries).**

| ESI 2002 | .630^**^ | |  | |  | |  | | |  | |  | | |  | |  | |  | |  | | |  |  |  |  |  |  |  |  |
| --- | --- | --- | --- | --- | --- | --- | --- | --- | --- | --- | --- | --- | --- | --- | --- | --- | --- | --- | --- | --- | --- | --- | --- | --- | --- | --- | --- | --- | --- | --- | --- |
| ESI 2005 | .604^**^ | | .656^**^ | |  | |  | | |  | |  | | |  | |  | |  | |  | | |  |  |  |  |  |  |  |  |
| EPI 2006 | .601^**^ | | .429^**^ | | .443^**^ | |  | | |  | |  | | |  | |  | |  | |  | | |  |  |  |  |  |  |  |  |
| EPI 2008 | .592^**^ | | .481^**^ | | .478^**^ | | .710^**^ | | |  | |  | | |  | |  | |  | |  | | |  |  |  |  |  |  |  |  |
| EPI 2010 | .509^**^ | | .423^**^ | | .404^**^ | | .581^**^ | | | .722^**^ | |  | | |  | |  | |  | |  | | |  |  |  |  |  |  |  |  |
| EPI 2012 | .509^**^ | | .410^**^ | | .410^**^ | | .558^**^ | | | .588^**^ | | .528^**^ | | |  | |  | |  | |  | | |  |  |  |  |  |  |  |  |
| EPI 2014 | .558^**^ | | .368^**^ | | .368^**^ | | .654^**^ | | | .626^**^ | | .550^**^ | | | .485^**^ | |  | |  | |  | | |  |  |  |  |  |  |  |  |
| EPI 2016 | .570^**^ | | .407^**^ | | .424^**^ | | .673^**^ | | | .638^**^ | | .572^**^ | | | .447^**^ | | .716^**^ | |  | |  | | |  |  |  |  |  |  |  |  |
| EPI 2018 | .519^**^ | | .372^**^ | | .374^**^ | | .636^**^ | | | .621^**^ | | .588^**^ | | | .489^**^ | | .696^**^ | | .730^**^ | |  | | |  |  |  |  |  |  |  |  |
| EPI 2020 | .563^**^ | | .393^**^ | | .399^**^ | | .692^**^ | | | .654^**^ | | .599^**^ | | | .509^**^ | | .742^**^ | | .760^**^ | | .760^**^ | | |  |  |  |  |  |  |  |  |
|  | ESI 2001 | | ESI 2002 | | ESI 2005 | | EPI 2006 | | | EPI 2008 | | EPI 2010 | | | EPI 2012 | | EPI 2014 | | EPI 2016 | | EPI 2018 | | |  |  |  |  |  |  |  |  |
|  | |  | | | |  | | |  | | | |  | | |  | | | |  | | |  | | |  | |  | |  | |
|  | | Degree of Correlation | | | | | | | | | | | | | | | | | | | | | | | | | | | | | |
|  | |  | |  | | | |  | | |  | | |  | | | |  | | | |  | | |  | |  | |  | |  |
|  | | weak positive | | moderate positive | | | | strong positive | | |  | | |  | | | |  | | | |  | | |  | |  | |  | |  |
|  | | 0.20 to 0.39 | | 0.40 to 0.59 | | | | 0.60 to 0.79 | | |  | | |  | | | |  | | | |  | | |  | |  | |  | |  |

Note:

**Correlation is significant at the 0.01 level (2 tailed)
